# PHF21B overexpression promotes cancer stem cell-like traits in prostate cancer cells by activating the Wnt/β-catenin signaling pathway

**DOI:** 10.1186/s13046-017-0560-y

**Published:** 2017-06-23

**Authors:** Qiji Li, Liping Ye, Wei Guo, Min Wang, Shuai Huang, Xinsheng Peng

**Affiliations:** 1 Department of Orthopaedic Surgery, the First Affiliated Hospital of Sun Yat-sen University, 510080 Guangzhou, Guangdong Province China; 2 Guangdong Provincial Key Laboratory of Orthopedics and Traumatology, Guangzhou, 510080 China; 3 Department of Experimental Research, State Key Laboratory of Oncology in Southern China, Sun Yat-sen University Cancer Center, 510060 Guangzhou, Guangdong Province China

**Keywords:** PHF21B, Prostate cancer, Cancer stem cell, Wnt/β-catenin signaling

## Abstract

**Background:**

PHF21B is newly identified to be involved in the tumor progression; however, its biological role and molecular mechanism in prostate cancer have not been defined. This study is aimed to study the role of PHF21B in the progression of prostate cancer.

**Methods:**

Real-time PCR, immunohistochemistry and western blotting analysis were used to determine PHF21B expression in prostate cancer cell lines and clinical specimens. The role of PHF21B in maintaining prostate cancer stem cell-like phenotype was examined by tumor-sphere formation assay and expression levels of stem cell markers. Luciferase reporter assay, western blot analysis, enzyme-linked immunosorbent assay and ChIP assay were used to determine whether PHF21B activates the Wnt/β-catenin signaling by transcriptionally downregulating SFRP1 and SFRP2.

**Results:**

Our results revealed that PHF21B was markedly upregulated in prostate cancer cell lines and tissues. High PHF21B levels predicted poorer recurrence-free survival in prostate cancer patients. Gain-of-function and loss-of-function studies showed that overexpression of PHF21B enhanced, while downregulation suppressed, the cancer stem cell-like phenotype in prostate cancer cells. Xenograft tumor model showed that silencing PHF21B decreased the ability of tumorigenicity in vivo. Notably, Wnt/β-catenin signaling was hyperactivated in prostate cancer cells overexpressing PHF21B, and mediated PHF21B-induced cancer stem cell-like phenotype. Furthermore, PHF21B suppressed repressors of the Wnt/β-catenin signaling cascade, including SFRP1 and SFRP2. These results demonstrated that PHF21B constitutively activated wnt/β-catenin signaling by transcriptionally downregulating SFRP1 and SFRP2, which promotes prostate cancer stem cell-like phenotype.

**Conclusions:**

Our results revealed that PHF21B functions as an oncogene in prostate cancer, and may represent a promising prognostic biomarker and an attractive candidate for target therapy of prostate cancer.

**Electronic supplementary material:**

The online version of this article (doi:10.1186/s13046-017-0560-y) contains supplementary material, which is available to authorized users.

## Background

Prostate cancer (PCa), one of the most lethal malignancies, constitutes 19% of all cancers diagnosed in men, with an estimated 161,360 new cases in the United States in 2017 [1]. While clinically localized PCa can be well controlled through radical prostatectomy, the 10-year recurrence rate in the intermediate and high risk localized PCa cases was 54% and 71%, respectively [2], leading to insurmountable obstacles for improving the survival rate [3, 4]. For patients with advanced prostate cancer, androgen deprivation therapy (ADT) has been the standard therapy. However, most patients relapse and develop castration-resistant prostate cancer within 18–24 months following ADT [5]. Cancer stem cells (CSCs), a small subpopulation of cancer cells, which possess self-renewal and multilineage differentiation potential, have been reported to play a critical role for PCa [6, 7]. CSCs are responsible for tumor initiation, chemotherapy resistance and become enriched after ADT treatment, which leads to poor outcomes and recurrence in PCa patients [8–13]. Therefore, unveiling the underlying molecular mechanism of prostate CSCs turns out to be the essential and profound topic for PCa treatment.

The Wnt/β-catenin signaling pathway, which is one of the most relevant pathways involved in CSCs, has been reported to be aberrantly activated in various types of cancers, including PCa [14–16]. Previous studies have shown that canonical Wnt signaling passway is associated with chemoresistance and recurrence of PCa [17–20]. Yun et al. reported that DAB2IP knockdown promotes PCa stem cell phenotypes through activation of Wnt/β-catenin signaling, and increases PCa cells chemoresistance [13]. Moreover, Huang et al. revealed that genetic variations of *adenomatous polyposis coli* (APC), tumor suppressor involved in the Wnt signaling pathway by regulating β-catenin degradation and nuclear export, are associated with recurrence of PCa following radical prostatectomy [18]. However, the underlying mechanism of how Wnt/β-catenin signaling regulates prostate CSCs remains to be elucidated.

Wnt/β-catenin signaling is initiated by the binding of Wnt to Frizzled (FZD) receptor and LRP-5/6, leading to the stabilization of cytosolic β-catenin [21, 22]. β-catenin then translocates to the nucleus and regulates the expression of a number of genes implicated in prostate CSCs regulation [13, 23]. On the other hand, there are several negative modulators involved in the Wnt/β-catenin signaling pathway for fine tuning the signaling. For instance, secreted Frizzled-related proteins (SFRPs), extracellular secreted Wnt inhibitors, can suppress Wnt ligands binding to frizzled receptor and block signal transduction [24]. Axin, GSK-3β and APC cause a robust suppression in the activity of Wnt/β-catenin signaling by forming a destruction complex and inducing β-catenin degradation [25]. Thus, understanding how these negative regulatory effects on the Wnt/β-catenin signaling pathway is clinically important for future development of PCa treatment.

Previous study has shown that proteins of the PHD zinc finger superfamily are capable of translocating to the nucleus and regulating transcription of genes, and involve in tumor progression in various types of cancers, including PCa [26–29]. High levels of PHF8 were associated with high Gleason grade and poor prognosis in PCa, and strengthened PCa cell migration and invasion in vitro [28]. Moreover, recently, Lapuk et al. found that PHF21A is differentially spliced in highly proliferative and aggressive neuroendocrine prostate cancer (NEPCa) versus PCa [29], in which these alternatively spliced genes were involved in EMT and important for cell shape and invasion. PHF21B, encoding the PHD finger protein 21B, is homologous to PHF21A and acts as a transcriptional repressor like PHF21A [30]. Previous study has reported that PHF21B was downregulated in head and neck squamous cell carcinomas (HNSCC), and reduced MDA-MB231 cells migration and colony formation in vitro [30]. However, the clinical implications and function of PHF21B in PCa have not been defined. In the present study, we found that PHF21B was significantly overexpressed in PCa and enhanced the stem cell-like traits of PCa cells by downregulating of negative modulators of the Wnt/β-catenin pathway, including SFRP1 and SFRP2. Therefore, our results suggest that PHF21B might serve as a novel therapeutic target in PCa.

## Methods

### Cell lines and cell culture

RWPE-1, PC-3, DU145, C4-2B, VCaP and LNCaP cells were obtained from the ATCC (Manassas, VA, USA). RWPE-1 cells were cultured in defined keratinocyte-SFM (1×) (Invitrogen, Carlsbad, CA, USA). PC-3, C4-2B and LNCaP cells were cultured in RPMI 1640 medium (Invitrogen) supplemented with10% FBS (Invitrogen), while DU145 and VCaP cells were cultured in Dulbecco's modified Eagle's medium (Invitrogen) supplemented with10% FBS.

### Patient information and tissue specimens

A total of 116 paraffin-embedded and archived PCa samples were collected for this study, which had been diagnosed histopathologically. Clinical information on the samples is summarized in detail in Additional file 1: Table S1. The fresh tissues including eight paired PCa tissues and adjacent non-tumor tissues were obtained from individuals who were diagnosed with PCa. All samples were collected from the First Affiliated Hospital of Sun Yat-sen University. Prior patient's consents were obtained to use these clinical specimens for research purposes. Our study was approved by the Ethics Committee of the First Affiliated Hospital of Sun Yat-sen University according to the 1975 Declaration of Helsinki.

### Plasmids, virus constructs and retroviral infection of target cells

A human PHF21B cDNA clone (EX-T2701-Lv105), as well as short hairpin RNA (shRNA) expression clone (HSH001525-CU6), was purchased from GeneCopoeia (Guangzhou, China). SMARTpool siRNA against human SFRP1, SFRP2, and β-catenin was purchased from RiboBio (Guangzhou, China). The reporter plasmids containing wild-type (CCTTTGATC; TOP flash) or mutated (CCTTTGGCC; FOP flash) TCF/LEF DNA binding sites were purchased from Upstate Biotechnology (New York, USA). Transfection of plasmids or siRNA was performed using the Lipofectamine 3000 reagent (Invitrogen, Carlsbad, CA, USA) according to the manufacturer’s instruction. Stable cell lines expressing PHF21B and PHF21B-shRNA were generated by retroviral infection and selected with 0.5 μg/ml puromycin for 10 days.

### RNA extraction, reverse transcription (RT) and real-time PCR

Total RNA was extracted from cultured cells and PCa tissues using the Trizol reagent (Invitrogen, Carlsbad, CA, USA) according to the manufacturer's instructions. A total of 2 μg of RNA was reverse transcribed to cDNA with M-MLV Reverse Transcriptase (Promega, Madison, US). qRT-PCR analysis was performed on an ABI Prism 7500 Sequence Detection System (Applied Biosystems, Texas, US). Expression data were normalized to the housekeeping gene GAPDH. Relative expression levels were calculated as 2^-[(Ct of gene) - (Ct of GAPDH)]^, in which Ct represents the threshold cycle for each transcript. The PCR primer sequences are listed in Additional file 2: Table S2.

### Western blotting

Western blotting was performed as described previously [31], using anti-PHF21B (ab70435, 1:1000; Abcam, Cambridge, UK), anti-β-catenin (#9562, 1:1000; Cell Signaling, Danvers, MA, USA), anti-phospho-β-Catenin (Ser33/37/Thr41) (#9561, 1:1000; Cell Signaling), and anti-p84 (ab102684, 1:500; Abcam) antibodies. Blotting membranes were stripped and re-probed with anti-α-tubulin antibody (T6199, 1:2000; Sigma-Aldrich, St. Louis, MO, USA) as a loading control. Nuclear extracts were prepared using the Nuclear Extraction kit (Active Motif), according to the manufacturer's instructions.

### Immunohistochemistry

Immunohistochemical (IHC) analysis was conducted to study protein expression in clinical specimens. The procedure was carried out similarly to previously described methods [32]. After deparaffinization, the sections were immunohistochemically stained using anti-PHF21B (HPA053834, 1:2500; Sigma-Aldrich), and anti-β-catenin antibody (#9562, 1:200; Cell Signaling). The degree of immunostaining offormalin-fixed, paraffin-embedded sections was examined and scored independently by two pathologists. The scores were determined by combining the proportion of positively stained tumor cells and the intensity of the IHC signals. Tumor cell proportions were scored as follows: 0, no positive tumor cells; 1, 1%-25% positive tumor cells; 2, 25%-50% positive cells; 3, 50%-75% positive tumor cells; and 4, >75% positive tumor cells. Staining intensity was graded according to the following criteria: 0, no staining; 1, weak staining (light yellow); 2, moderate staining (yellow brown); and 3, strong staining (brown). The staining index (SI) was calculated as the staining intensity score × the proportion of positive tumor cells (ranging from 0 to 12). We evaluated protein expression by determining the SI. Samples with an SI ≥ 6 were classified as showing high expression, while samples with an SI < 6 were classified as showing low expression.

### Enzyme-linked immunosorbent assay (ELISA)

Using a commercially available SFRP1 (E95880Hu) and SFRP2 (E95879Hu) ELISA Kit (USCN, Wuhan, China), the concentration of SFRP1 and SFRP2 in the conditioned media (CM) derived from the cells was determined. ELISA was performed according to the directions of the manufacturer.

### Sphere formation assay

Cells (500/well) were seeded into 6-well ultra-low cluster plates (Corning, NY) and cultured in DMEM/F12 serum-free medium (Invitrogen) supplemented with 2% B27 (Invitrogen), 20 ng/ml EGF (PeproTech, Rocky Hill, NJ, USA), 20 ng/ml bFGF (PeproTech), 0.4% BSA (Sigma-Aldrich), and 5 μg/ml insulin (Sigma-Aldrich). After 10 days, the number of spheres was counted, and images of the spheres were photographed.

### Luciferase activity assay

Thirty thousand cells were cultured in triplicate in 48-well plates for 24 h. Then, 100 ng of luciferase reporter plasmids containing the SFRP1 and SFRP2 promoter, TOP-Flash or FOP-Flash luciferase reporter, plus 3 ng of pRL-TK Renilla plasmid (Promega), were transfected into cells using the Lipofectamine 3000 reagent (Invitrogen, Carlsbad, CA, USA) according to the manufacturer’s recommendations. Luciferase and Renilla signals were measured 36 h after transfection using the Dual Luciferase Reporter Assay Kit (Promega) according to the manufacturer’s protocol.

Specific primers used for promoter luciferase reporter were presented in Additional file 3: Table S3.

The promoter sequences of SFRP1/2 were obtained from the UCSC Genome Browser (http://genome.ucsc.edu) referring the enrichment of H3K27Ac mark (often found near active regulatory elements) on seven cell lines as determined by ChIP-seq assays provided by the website. Each of the cell lines including GM12878, H1-hESC, HSMM, HUVEC, K562, NHEK, NHLF in this track is consistent across the ENCODE (Encyclopedia of DNA Elements) Regulation super-track.

### Chromatin immunoprecipitation (ChIP)

The SimpleChIP Enzymatic Chromatin IP Kit with magnetic beads (#9003, Cell Signaling, Danvers, MA, USA) was used according to the manufacturer's protocol. Cells (4 × 10^6^) transfected with Flag-tagged PHF21B (EX-T2701-Lv102; GeneCopoeia, Guangzhou, China) in a 100 mm culture dish were treated with formaldehyde to cross-link proteins to DNA. 1× Glycine was added to terminate cross-linking. A total of 5 μg of anti-FLAG antibody (SAB4200071; Sigma, St Louis, MO, USA),) or anti-IgG antibody was incubated with 10 μg of sheared chromatin overnight at 4 °C. ChIP-grade protein G magnetic beads were added and incubated for 2 h at 4 °C with rotation. Immunoprecipitated chromatin was then washed with low- and high-salt ChIP buffer. After reverse cross-linking of protein/DNA complexes to free DNA, PCR was performed. Primers used for ChIP are presented in Additional file 4: Table S4–S5.

### Xenograft tumor model

All experimental procedures were approved by the Institutional Animal Care and Use Committee of Sun Yat-Sen University. Male BALB/c-nude mice (4–6 weeks old, 18–20 g) were purchased from the Slac-Jingda Animal Laboratory (Hunan, China) and housed in barrier facilities on a 12-h light/dark cycle. Mice were randomly allocated into groups (*n* = 6 per group). The indicated cells at three doses (1 × 10^6^, 1 × 10^5^, and 1 × 10^4^) were inoculated subcutaneously with Matrigel (final concentration of 25%) into the inguinal folds of the mice. Tumor volume was determined using an external caliper and calculated using the equation (L × W^2^)/2. After mice were sacrificed, the tumors were excised, weighed and subjected to pathologic examination. The frequency of tumor-initiating cells (TICs) was calculated using the Extreme Limiting Dilution Analysis (ELDA) program (http://bioinf.wehi.edu.au/software/elda/) [33].

### Statistical analysis

All statistical analyses were carried out using the SPSS 17.0 statistical software package (SPSS Inc, Chicago, IL, USA). The relationship between PHF21B expression and the clinicopathological characteristics was tested by the *χ* ^2^ test. Bivariate correlations between study variables were calculated using Spearman's rank correlation coefficients. Survival curves were plotted with the Kaplan-Meier method and compared by the log-rank test. Other comparisons were analyzed by unpaired two-sided independent Student’s *t*-test without equal variance assumption. *P* < 0.05 was considered statistically significant.

## Results

### Expression of PHF21B is upregulated in PCa cell lines and tissues

**Fig. 1:**
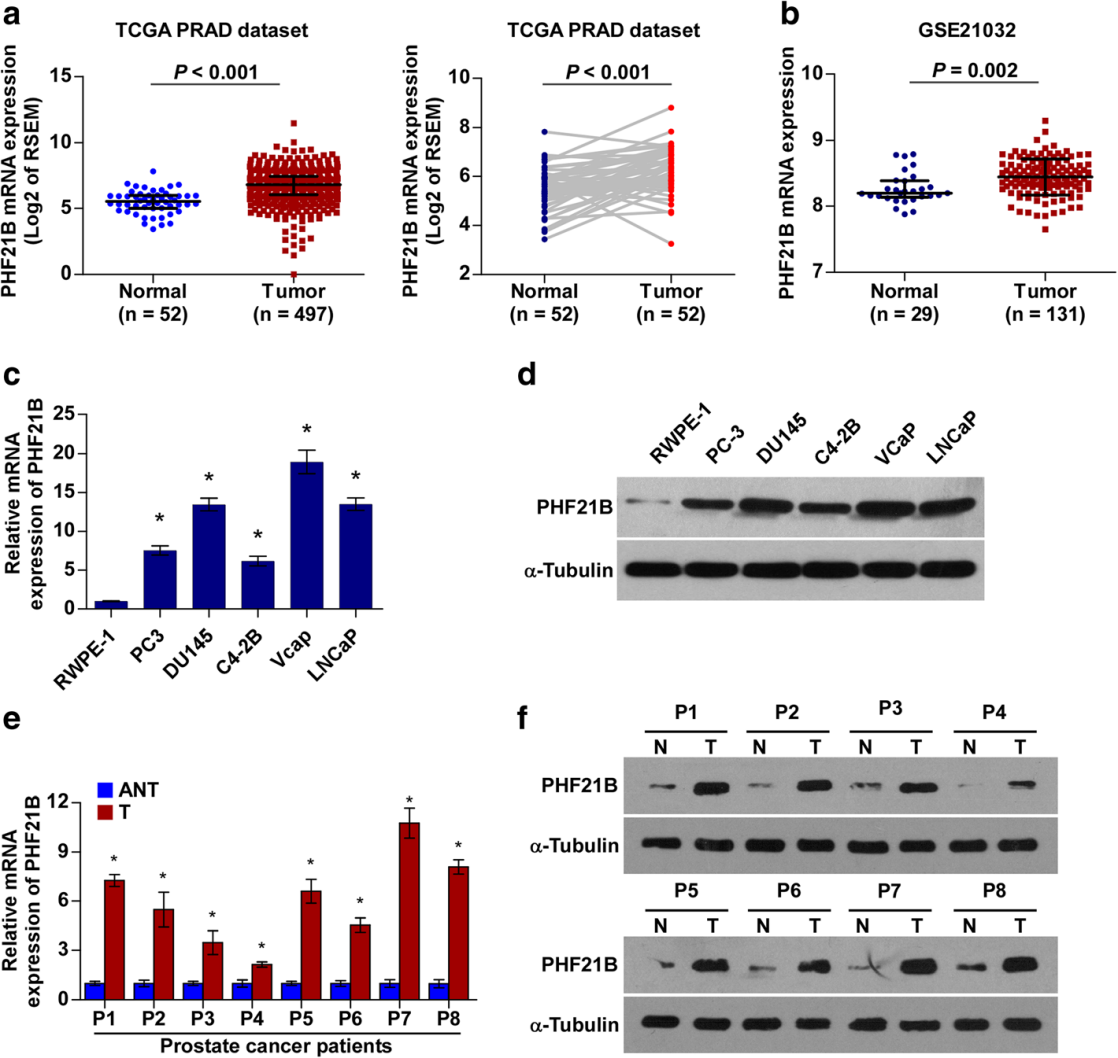
PHF21B is overexpressed in PCa cell lines and tissues. **a** PHF21B mRNA levels in PCa tissues were assessed by analyzing the TCGA prostate adenocarcinoma mRNA dataset (PRAD, Normal, *n* = 52; Tumor, *n* = 497 (*Left panel*) and 52 paired adjacent non-tumor tissues (Normal) and PCa tissues (Tumor) (*Right panel*). Data were acquired from the TCGA data portal (https://portal.gdc.cancer.gov/). **b** PHF21B mRNA levels in PCa tissues were assessed by analyzing the GSE21032 PCa mRNA dataset (Normal prostate tissue (Normal), *n* = 29; Clinically localized primary PCa tissue (Tumor), *n* = 131; Data were acquired from the Gene Expression Omnibus (http://www.ncbi.nlm.nih.gov/geo/)). **c**-**d** Real-time PCR (**c**) and Western blotting (**d**) analyses to detect PHF21B expression in normal human prostate epithelial cell (RWPE-1) and cultured PCa cell lines. **e**-**f** Real-time PCR (**e**) and Western blotting (**f**) analyses of PHF21B expression in paired primary PCa tissues (T) and the matched adjacent non-tumor tissues (ANT) from eight prostate cancer patients (P1-P8). mRNA expression was normalized to GAPDH, and α-tubulin was used as a protein loading control. Data represent the mean ± SD of three independent experiments; *P < 0.05

By analysis of the RNA expression data from The Cancer Genome Atlas (TCGA), we found that PHF21B levels were significantly upregulated in human PCa tissues (*n* = 497) compared with that in normal prostate tissues (*n* = 52) (*P* < 0.001) (Fig. 1a). Overexpression of PHF21B in PCa was confirmed by analyzed prostate cancer GEO dataset GSE21032 (Fig. 1b). We further verified this result in PCa cell lines and paired tissues using realtime PCR and western blotting analysis. As shown in Fig. 1c-f, PHF21B expression levels were differentially increased in five PCa cell lines than that in normal prostate epithelial cell (RWPE-1), and in 8 PCa tissues (T) compared to that in the paired adjacent normal tissues (ANT). Collectively, these results suggested that PHF21B was upregulated and might be involved in human PCa progression.

### Upregulation of PHF21B correlates with progression and poor prognosis in PCa

**Fig. 2:**
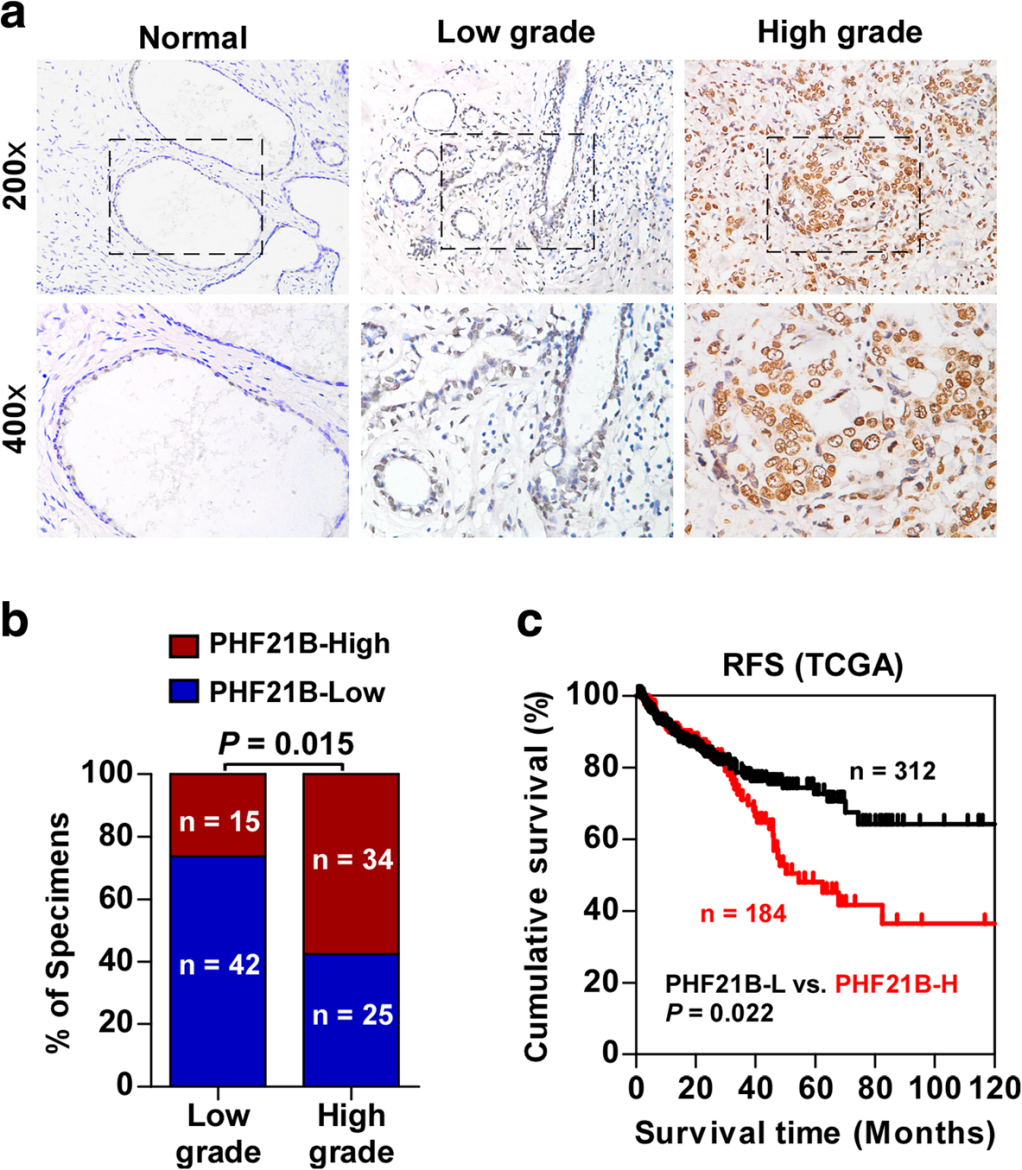
Upregulation of PHF21B correlates with progression and poor prognosis in PCa. **a** Representative IHC analyses of PHF21B expression in normal prostate tissue, low- and high-grade PCa specimens. **b** Percentage of PCa specimens showing low- and high-grade PCa relative to the level of PHF21B. **c** Kaplan-Meier analysis of recurrence-free survival curves for PCa patients with low PHF21B expression (PHF21B-L; *n* = 312) versus high PHF21B expression (PHF21B-H; *n* = 184) by analyzing the TCGA prostate adenocarcinoma dataset (*P* = 0.022, log-rank test)

To investigate the clinical significance of upregulation of PHF21B in PCa, PHF21B protein expression was examined in 116 paraffin-embedded, archived PCa tissues using IHC. As shown in Fig. 2a-b and Additional file 1: Table S1, expression of PHF21B correlated significantly with tumor stage (*P* = 0.011), total PSA level (*P* = 0.005) and Gleason score (*P* = 0.001) in PCa. We further assessed the correlation between PHF21B expression and the prognosis of the patient by analyzing the TCGA prostate adenocarcinoma (PRAD) dataset. Notably, Kaplan-Meier survival analysis revealed that patients with high PHF21B expression had poorer recurrence-free survival (RFS) than patients with low PHF21B expression in TCGA (Fig. 2d, *P* = 0.022), indicating that PHF21B may have potential as an independent prognostic marker in PCa.

### PHF21B regulates the stem cell-like phenotype in PCa cells

**Fig. 3:**
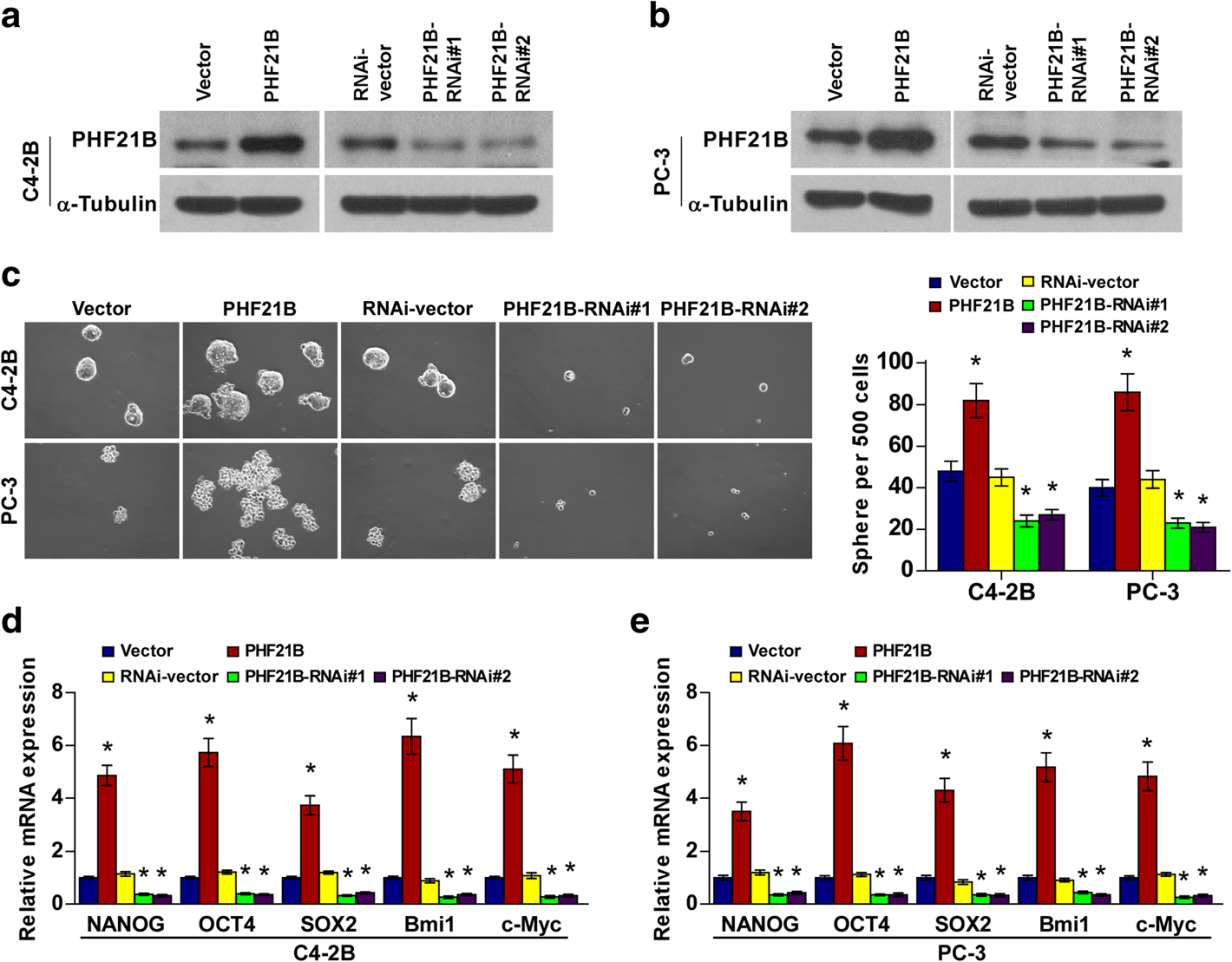
PHF21B regulates the stem cell-like phenotype in PCa cells in vitro. **a**-**b** Western blotting of PHF21B protein expression in the constructed C4-2B (**a**) and PC-3 (**b**) cells. α-Tubulin was used as a loading control. **c** Representative images of spheres formed by the indicated cells. Histograms show the mean number of spheres. **d**-**e** Real-time PCR analysis of the expression of pluripotency-associated markers, including NANOG, OCT4, SOX2, Bmi1, and c-Myc, in the indicated cells. The expression of different stem cell markers in different groups was normalized to that in the PHF21B vector control group. Error bars represent the means ± SD of 3 independent experiments. * *P* < 0.05. Original magnification, **c**, ×100

It has been noted that obtaining a stem-like phenotype of PCa cells is critical for malignant tumor and high frequency of recurrence [8, 9]. We then investigated the role of PHF21B upregulation in the self-renewal ability of PCa cells. PCa cell lines C4-2B and PC-3 were engineered to overexpress or silence PHF21B via lentivirus infection (Fig. 3a-b). Sphere-formation assays showed that upregulating PHF21B increased, while downregulating PHF21B decreased, the number and size of tumor spheroids in C4-2B and PC-3 cells (Fig. 3c). In addition, pluripotency-associated markers, including NANOG, OCT4, SOX2, Bmi1 and c-Myc, showed significantly higher mRNA expression in PHF21B-transduced cells but much lower in PHF21B-silenced cells than in the control group (Fig. 3d-e). Thus, our results indicated that PHF21B promoted the stem cell-like phenotype in PCa.

### PHF21B promotes PCa cells tumorigenicity in vivo

**Fig. 4:**
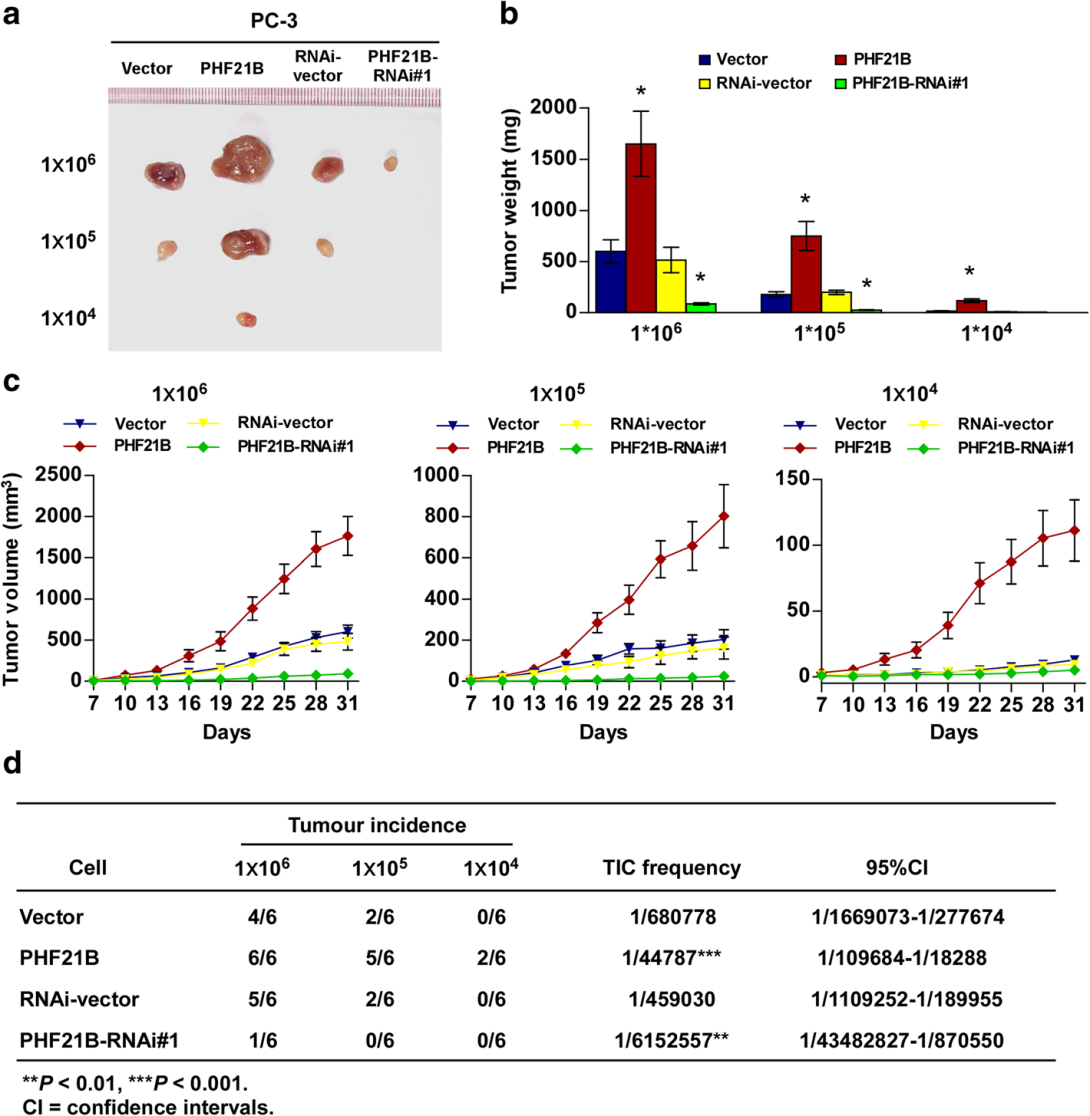
PHF21B promotes PCa tumorigenicity in vivo. **a** Xenograft model in nude mice (*n* = 6/group). Tumors formed by PHF21B-overexpressing PC-3 cells were larger than vector-control tumors in the group receiving 1 × 10^6^ cells. Conversely, tumors formed by PHF21B-silenced cells were smaller than tumors formed by control cells. Only PHF21B-overexpressing cells formed visible tumors following the implantation of 1 × 10^4^ cells. Representative images of the tumors are shown. **b** Histograms showing the mean tumor weights of each group. **c** Growth curves of tumor formation after implantation. Mean tumor volumes are plotted. **d** Effect of PHF21B on the tumorigenic capacity of PC-3 cells. The frequency of TICs was calculated using the ELDA program. Error bars represent the means ± SD. * *P* < 0.05. *P* values were based on *t*-test unless otherwise indicated

To further confirm the effect of PHF21B on PCa cell stemness, we subcutaneously inoculated different numbers of cells mixed with matrigel into the inguinal folds of BALB/c-nude mice. As shown in Fig. 4a-d, the tumors formed by PHF21B-transduced PCa cells, at both amount of 1 × 10^6^, 1 × 10^5^ or 1 × 10^4^ cells, were dramatically larger than the vector control tumors. Conversely, PHF21B-silenced cells formed much smaller tumors and presented lower rates of tumorigenicity (Fig. 4a-d). Importantly, only PHF21B-transduced cells could form tumors when 1 × 10^4^ cells were implanted (Fig. 4d). These results indicated that PHF21B strongly promoted PCa tumorigenicity in vivo.

### PHF21B activates Wnt/β-catenin signaling pathway

**Fig. 5:**
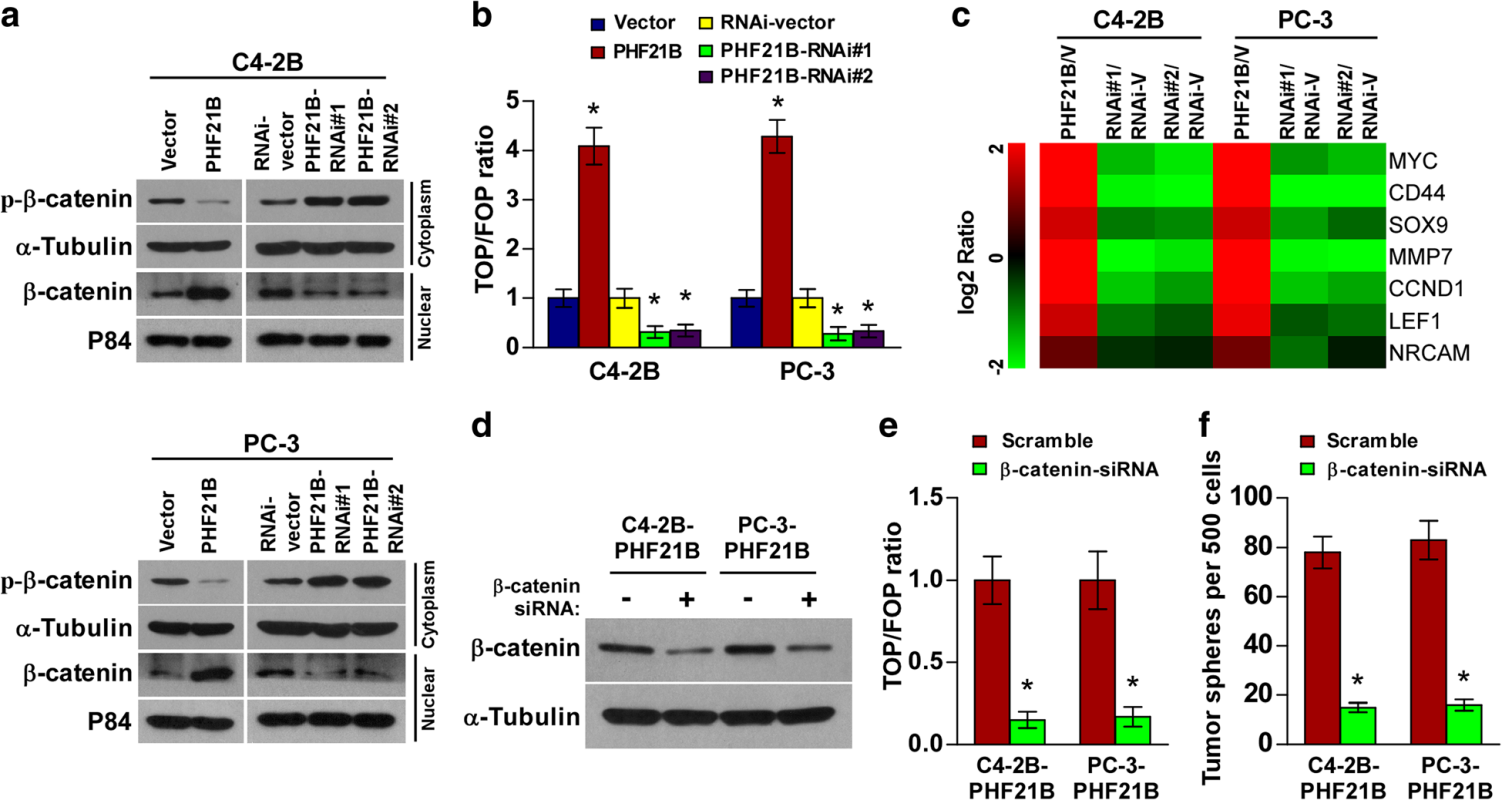
PHF21B activates Wnt/β-catenin signaling pathway. **a** PHF21B overexpression promoted the stabilization of cytosolic β-catenin, and increased β-catenin nuclear accumulation in indicated cells, as confirmed by western blot analysis. α-Tubulin and P84 were used as loading controls. **b** Luciferase-reported TCF/LEF transcriptional activity in the indicated cells. **c** Fold change of indicated genes mRNA expression in Real-time PCR analysis comparing cells overexpressing PHF21B versus Vector (V) or PHF21B-RNAi versus RNAi-vector (RNAi-V). **d** Knockdown of β-catenin with specific siRNA in PHF21B-overexpressing cells, as confirmed by western blot analysis. **e** Luciferase-reported TCF/LEF transcriptional activity in the indicated cells. **f** Histograms showing the mean number of spheres formed by the indicated cells. Each bar represents the mean ± SD of three independent experiments.*P < 0.05

Since Wnt/β-catenin signaling is one of the most important pathways in maintaining stem cell phenotype and frequently activates in PCa [16, 23], we then examined the role of PHF21B in Wnt/β-catenin signaling pathway. As shown in Fig. 5a, cellular fractionation and western blot analysis revealed that overexpression of PHF21B increased, while silencing of PHF21B reduced nuclear β-catenin expression. In addition, we found that PHF21B overexpression significantly increased, but silencing PHF21B reduced the TCF/LEF activities in PCa cell lines (Fig. 5b). Consistently, the expression of MYC, CD44, SOX9, MMP7, CCND1, LEF1, and NRCAM, which are well-characterized downstream targets of Wnt/β-catenin, were significantly increased in PHF21B-transduced cells but reduced in PHF21B-silenced cells (Fig. 5c). Collectively, our results suggested that PHF21B activated Wnt/β-catenin signaling pathway in PCa.

We further investigated the functional significance of Wnt/β-catenin signaling activation in PHF21B-mediated self-renewal of PCa cells by silencing β-catenin in PHF21B-overexpressing PCa cell lines (Fig. 5d). As expected, the stimulatory effect of PHF21B on TOP/FOP luciferase reporter activity was impaired by silencing β- catenin (Fig. 5e). Moreover, sphere formation assays indicated that silencing β- catenin abrogated the promotive effects of PHF21B on self-renewal of PCa cells (Fig. 5f). Thus, these results reveal that activation of Wnt/β-catenin signaling was essential for PHF21B-promoted stem cell-like phenotype in PCa.

### PHF21B suppresses the repressors of Wnt/β-catenin signaling pathway

**Fig. 6:**
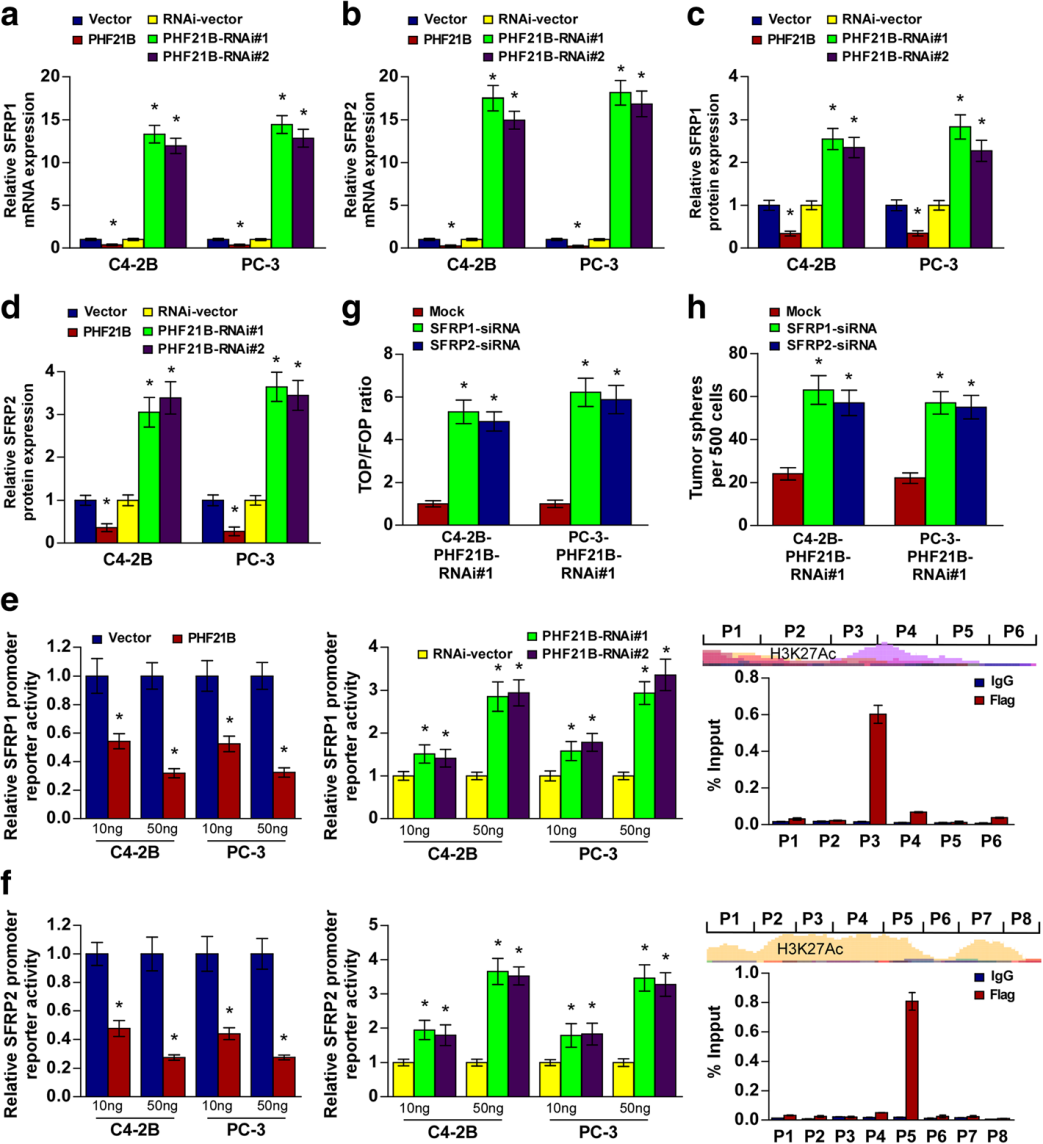
PHF21B suppresses the repressors of Wnt/β-catenin signaling pathway. **a**-**b** Real-time PCR analysis of SFRP1(**a**) and SFRP2 (**b**) expression in the indicated cells. **c**-**d** ELISA analysis of SFRP1(**c**) and SFRP2 (**d**) expression in the indicated cells. **e**-**f** *Left panel*: Luciferase activity assays in PC-3 and C4-2B cells showed trans-inactivation of the SFRP1(**e**) and SFRP2(**f**) promoters by PHF21B overexpression and trans-activation by PHF21B silencing. *Right panel*: ChIP enrichment assay confirmed that Flag-tagged PHF21B binds to the promoter of SFRP1 (**e**) and SFRP2 (**f**); IgG was used as a negative control. Schematic illustration of the indicated nucleotide regions of the SFRP1 and SFRP2 promoters. H3K27Ac enrichment, indicating high transcription activity, is observed in the promoter elements according to Genome Browser Gateway website. **g** Luciferase-reported TCF/LEF transcriptional activity in the indicated cells. **h** Histograms showing the mean number of spheres formed by the indicated cells. Each bar represents the mean ± SD of 3 independent experiments.*P < 0.05

PHF21B, with a PHD zinc finger domain, localizes to the nucleus and acts as a transcriptional repressor like PHF21A [30]. To investigate the mechanism by which PHF21B activates Wnt/β-catenin signaling passway, we examined whether PHF21B regulates the transcription of negative modulators in Wnt/β-catenin signaling. Indeed, SFRP1 and SFRP2 mRNA and protein expression were both downregulated in PHF21B-transduced PCa cells but upregulated in PHF21B-silenced PCa cells compared with control cells, revealing that SFRP1 and SFRP2 might be transcriptionally downregulated by PHF21B in PCa cells (Fig. 6a-d). As expected, luciferase reporter assays revealed that overexpression of PHF21B attenuated, whereas downregulation of PHF21B activated, the luciferase activity of SFRP1 and SFRP2 promoters in PCa cells in a dose-dependent manner (Fig. 6e-f). Furthermore, ChIP assays revealed that PHF21B was capable of binding to different fragment regions within the SFRP1 and SFRP2 promoters (Fig. 6e-f), suggesting that PHF21B transcriptionally downregulated SFRP1 and SFRP2 expression by directly targeting the SFRP1 and SFRP2 promoters.

In addition, individual silencing of SFRP1 or SFRP2 potently rescued the TOP/FOP luciferase reporter activity and self-renewal ability in PHF21B-silenced cells (Fig. 6g-h and Additional file 5: Figure S1a-b), revealing that SFRP1 and SFRP2 were functional effectors of PHF21B on regulating Wnt/β-catenin signaling and stem cell-like phenotype in PCa. Moreover, as shown in the Figure S1a-b, SFRP1 was not significantly affected by SFRP2 siRNA, and SFRP1 siRNA had no effect on the SFRP2 levels, in PHF21B-RNAi#1 PCa cells, suggesting that either SFRP1 or SFRP2 siRNA rescued TOPflash activity and self-renewal ability in PHF21B-silenced cells was not attributed to the siRNA cross-reaction. Collectively, our results suggested that PHF21B activated Wnt/β-catenin signaling to enhance stem cell-like phenotype in PCa by transcriptionally downregulated SFRP1 and SFRP2.

### Clinical relevance of PHF21B and β-catenin in human PCa

**Fig. 7:**
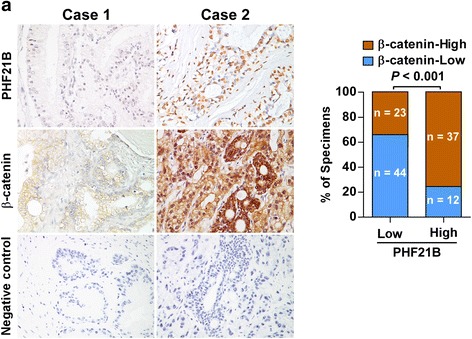
Clinical relevance of PHF21B and β-catenin in human PCa. **a** *Left panel*: PHF21B expression was positively correlated with β-catenin expression levels in PCa tissue specimens. Two representative cases are shown. Negative controls for IHC analysis by substitution of the primary antibody with PBS were provided. Original magnification, ×400. *Right panel*: Percentage of PCa specimens showing low or high PHF21B expression relative to the level of β-catenin

IHC analysis of PCa tissue specimens showed that PHF21B was positively correlated with β-catenin expression levels (*P* < 0.001, Fig. 7a). Collectively, these data further support the notion that PHF21B upregulation in PCa activates canonical Wnt/β-catenin signaling, ultimately leading to PCa progression.

## Discussion

Despite many recent advances, PCa continues to be the leading cause of male cancer-related mortality and morbidity. Most of PCa patients diagnosed with clinically localized disease can be successfully controlled by definitive treatments-radical prostatectomy or radiotherapy alone or in combination with androgen ablation. However, the 10-year recurrence rate is very high [2, 34]. In addition, almost all advanced PCa patients relapse and progress to lethal castration-resistant disease after initial response to androgen ablation [5]. The CSC has been proposed to be responsible for the high rate of relapse and subsequent resistance of advanced PCa to ADT. CSCs have been identified in many solid malignancies, including PCa [6], and have significant clinical implications, as targeting the CSC population may be essential in preventing the recurrence and progression of castration-resistant PCa [13, 17, 35].

Herein, we found that PHF21B acted as a potent CSC-promoting factor in PCa. Overexpression of PHF21B promoted, while downregulation reduced, the self-renewal of PCa cells and the expression of pluripotency-associated markers, including NANOG, OCT4, SOX2, Bmi1 and c-Myc. In addition, inhibition of PHF21B potentially reduced PCa cells tumorigenicity in vivo. Thus, our results suggested that upregulation of PHF21B is involved in the malignant progression of PCa, and proposed that PHF21B might be a potential therapeutic target for human PCa.

Wnt/β-catenin signaling pathway plays a key role in the regulation of CSC self-renewal and is aberrantly activated in PCa [14, 23]. In addition, the functions of Wnt/β-catenin signaling in PCa development and progression have been well documented. Mesd, a general Wnt inhibitor, suppressed Wnt/β-catenin signaling induced by Wnt1 in PC-3 cells, and inhibited PC-3 cells tumorigenicity in vivo [36]. Unlike in colon cancer, the mutations in β-catenin are rare, but the expression and/or nuclear localization of β-catenin are often abnormal in PCa, suggesting a constitutive activation of Wnt/β-catenin signaling [20, 37, 38]. However, how cancer cells escape the negative regulation by Wnt/β-catenin signaling cascade, leading to constitutively activated β-catenin/TCF remains unclear. Herein, we found that PHF21B was upregulated in PCa and induced activation of Wnt/β-catenin signaling via directly transcriptionally downregulating SFRP1 and SFRP2. Therefore, our findings not only confirmed activation of Wnt/β-catenin pathway contributes to the malignant behavior of PCa, but also revealed a novel mechanism for activation of Wnt/β-catenin pathway involving PHF21B in PCa.

In our result, we found that PHF21B was positively correlated with β-catenin expression levels in PCa tissue specimens (*P* < 0.001) (Fig. 7a). However, we also observed that 12 of 49 cases (~24%) of PCa with high PHF21B exhibited low protein level of β-catenin. Previous studies have found that human cancers exhibit substantial intra-tumor heterogeneity in transcriptional identities and gene expression [39]. Therefore, the mechanisms by which β-catenin protein is reduced in PCa tissues with high PHF21B might be attributed to tumor heterogeneity. Moreover, there might be some transcriptional cofactors [40] that bind PHF21B or block associations between PHF21B and the SFRP1 and SFRP2 promoters, under certain circumstances, thus inhibit trans-inactivation of SFRP1 and SFRP2. In addition, recruitment of chromatin-modifying factors might be another reason [41]. In this respect, further investigation of the mechanisms by which β-catenin protein is reduced in PCa with high PHF21B will eventually lead to the development of a new therapeutic strategy for the treatment of PCa.

PHF21B has been reported to be downregulated in HNSCC, and overexpression of PHF21B reduced MDA-MB231 cells migration and colony formation in vitro, suggesting it might act as a tumor suppressor in HNSCC and breast cancer [30]. However, in the current study, PHF21B was significantly upregulated in PCa, and enhanced the stem cell-like traits of PCa cells by activating the Wnt/β-catenin pathway. Therefore, the PHF21B may display both tumor suppressive and oncogenic properties depending on the tissue types and cellular conditions.

## Conclusions

In summary, this study provided the first report of the expression of PHF21B in PCa cell lines and tissues, and demonstrated that upregulation of PHF21B may contribute to the progression and poor prognosis of human PCa. Moreover, these data suggests that PHF21B functioned as an oncogene in PCa and may represent a promising prognostic biomarker for PCa. Therefore, this study elucidated the precise role of PHF21B in PCa, and may help to identify more effective therapeutic strategies for PCa.

## Additional files

Additional file 1: Table S1. Correlation between PHF21B expression and clinicopathological characteristics of prostate cancer patients. (DOC 38 kb)

Additional file 2: Table S2. Real-time PCR primers. (DOC 37 kb)

Additional file 3: Table S3. The primers used for promoter luciferase reporter. (DOC 29 kb)

Additional file 4: Table S4-S5. The primers used for ChIP assay. (DOC 50 kb)

Additional file 5: Figure S1. SFRP1 and SFRP2 protein expression in the indicated cells. (A-B) Enzyme-linked immunosorbent assay (ELISA) analysis of protein levels of SFRP1 (A) and SFRP2 (B) in the supernatants of PHF21B-RNAi#1-C4-2B and -PC-3 cells treated with SFRP1 or SFRP2 siRNA. Error bars represent the means ± SD of 3 independent experiments. *P < 0.05. Not significant, n.s.. (TIF 103 kb)

## Abbreviations

ADT: Androgen deprivation therapy
APC: Adenomatous polyposis coli
CSC: Cancer stem cell
ELDA: Extreme Limiting Dilution Analysis
ELISA: Enzyme-linked immunosorbent assay
PCa: Prostate cancer
SFRPs: Frizzled-related proteins
shRNA: Short hairpin RNA
TCGA: The Cancer Genome Atlas.
