# Supplementary material for: PHF21B overexpression promotes cancer stem cell-like traits in prostate cancer cells by activating the Wnt/β-catenin signaling pathway
Source: J Exp Clin Cancer Res. 2017 Jun 23;36:85. doi: 10.1186/s13046-017-0560-y (PMC5481925; doi:10.1186/s13046-017-0560-y)
Supplement: Supplementary file 1 — Correlation between PHF21B expression and clinicopathological characteristics of prostate cancer patients. (DOC 38 kb) [file 13046_2017_560_MOESM1_ESM.doc]

**Table S1.** Correlation between PHF21B expression and clinicopathological characteristics of prostate cancer patients.

| **Characteristics** | | **Total** | **PHF21B** | | ***p* value** |
| --- | --- | --- | --- | --- | --- |
|  |  |  | **Low** | **High** |  |
| Age (years) | ≤ 73 | 49 | 29 | 20 | 0.790 |
|  | > 73 | 67 | 38 | 29 |  |
| Tumor stage | ≤ T2b | 81 | 53 | 28 | 0.011 |
|  | ≥ T3a | 35 | 14 | 21 |  |
| Total PSA (ng/ml) | ≤ 10 | 30 | 24 | 6 | 0.005 |
|  | >10 | 86 | 43 | 43 |  |
| Gleason score | ≤ 7 | 57 | 42 | 15 | 0.001 |
|  | >7 | 59 | 25 | 34 |  |
