# Supplementary material for: PHF21B overexpression promotes cancer stem cell-like traits in prostate cancer cells by activating the Wnt/β-catenin signaling pathway
Source: J Exp Clin Cancer Res. 2017 Jun 23;36:85. doi: 10.1186/s13046-017-0560-y (PMC5481925; doi:10.1186/s13046-017-0560-y)
Supplement: Supplementary file 2 — Real-time PCR primers. (DOC 37 kb) [file 13046_2017_560_MOESM2_ESM.doc]

**Table S2.** Real-time PCR primers.

| **Name** | **Sequence (5’to 3’)** |
| --- | --- |
| PHF21B-Up | CTCAGGGAGCTCAGGCCA |
| PHF21B-Down | CTTCTCTTCCGCTCCTGTCG |
| SFRP1-Up | AGATGCTTAAGTGTGACAAGTTCCC |
| SFRP1-Down | TGGCCTCAGATTTCAACTCGT |
| SFRP2-Up | ATGATGATGACAACGACATAATG |
| SFRP2-Down | ATGCGCTTGAACTCTCTCTGC |
| NANOG-Up | TCCAACATCCTGAACCTCAGCTA |
| NANOG-Down | AGTCGGGTTCACCAGGCATC |
| OCT4-Up | GTCCGAGTGTGGTTCTGTA |
| OCT4-Down | CTCAGTTTGAATGCATGGGA |
| SOX2-Up | GTGAGCGCCCTGCAGTACAA |
| SOX2-Down | GCGAGTAGGACATGCTGTAGGTG |
| Bmi1-Up | TCGTTGTTCGATGCATTTCT |
| Bmi1-Down | CTTTCATTGTCTTTTCCGCC |
| c-Myc-Up | CCTCCACTCGGAAGGACTATC |
| c-Myc-Down | TGTTCGCCTCTTGACATTCTC |
| GAPDH-Up | GCACCGTCAAGGCTGAGAAC |
| GAPDH-Down | TGGTGAAGACGCCAGTGGA |
