# Supplementary material for: PHF21B overexpression promotes cancer stem cell-like traits in prostate cancer cells by activating the Wnt/β-catenin signaling pathway
Source: J Exp Clin Cancer Res. 2017 Jun 23;36:85. doi: 10.1186/s13046-017-0560-y (PMC5481925; doi:10.1186/s13046-017-0560-y)
Supplement: Supplementary file 3 — The primers used for promoter luciferase reporter. (DOC 29 kb) [file 13046_2017_560_MOESM3_ESM.doc]

**Table S3.** The primers used for promoter luciferase reporter.

| **Name** | **Sequence (5’to 3’)** |
| --- | --- |
| SFRP1-luc-Up | AGGTACCGAGCTCTTACGCGTGCCGATGTCCACGCACTGAGGTG |
| SFRP1-luc-Down | TGCAGTCGGGGCGGCAGATCTAAGTGTCCTCCCAGAGCTAATACC |
| SFRP2-luc-Up | AGGTACCGAGCTCTTACGCGTGCCAGTGCGAGGCGAGGAAGAG |
| SFRP2-luc-Down | TGCAGTCGGGGCGGCAGATCTCAGCCAGACCAGCTTCGG |
