# Supplementary material for: PHF21B overexpression promotes cancer stem cell-like traits in prostate cancer cells by activating the Wnt/β-catenin signaling pathway
Source: J Exp Clin Cancer Res. 2017 Jun 23;36:85. doi: 10.1186/s13046-017-0560-y (PMC5481925; doi:10.1186/s13046-017-0560-y)
Supplement: Supplementary file 4 — The primers used for ChIP assay. (DOC 50 kb) [file 13046_2017_560_MOESM4_ESM.doc]

**Table S4.** The primers of SFRP1 used for ChIP assay.

| **SFRP1-ChIP-primer** | **Sequence (5’to 3’)** |
| --- | --- |
| P1-Up | GATGTCCACGCACTGAGGTG |
| P1-Down | AGGTCCCTGGAAGTTTGCG |
| P2-Up | CCGCAAACTTCCAGGGAC |
| P2-Down | GTGGTAACGAGTGCGGCTC |
| P3-Up | GCGAGCCGCACTCGTTAC |
| P3-Down | CGAGTTAATTACAGCGTCCGC |
| P4-Up | CAGGGCGGACGCTGTAAT |
| P4-Down | GCCTGGCTAACATGGTGAAAC |
| P5-Up | TTAGTAGAGACGGGGTTTCACCA |
| P5-Down | GCCAGGCCCACTGGGTAT |
| P6-Up | GAGGTCTGCTTGTAAATACCCAGT |
| P6-Down | AAGTGTCCTCCCAGAGCTAATACC |

**Table S5.** The primers of SFRP2 used for ChIP assay.

| **SFRP2-ChIP-primer** | **Sequence (5’to 3’)** |
| --- | --- |
| P1-Up | AGTGCGAGGCGAGGAAGAG |
| P1-Down | CCACCCTCCAGATTTGCATAA |
| P2-Up | TTTATGCAAATCTGGAGGGTG |
| P2-Down | CCACTCATGCCTGGCAAC |
| P3-Up | TCGCGACCAGGTACTGGC |
| P3-Down | AGCACACCGACAGGCTGC |
| P4-Up | GAATCTCCAGCCACCGTTCA |
| P4-Down | GCCACCAGTCTTTACAAACAGTG |
| P5-Up | CCAGGGCACTGTTTGTAAAGACT |
| P5-Down | AGCGAAGGCCTCCTGTCC |
| P6-Up | TTGCCTAATTGTCCCTAGTCTCC |
| P6-Down | GACCGCAAAGTGTGCCTGA |
| P7-Up | AAGTTATCAGGCACACTTTGCG |
| P7-Down | TTCTGTTTCCTTTGTGGTTTCATT |
| P8-Up | CTTTGCAATGAAACCACAAAGG |
| P8-Down | CAGCCAGACCAGCTTCGG |
