# Supplementary figures and images for: PHF21B overexpression promotes cancer stem cell-like traits in prostate cancer cells by activating the Wnt/β-catenin signaling pathway
Source: J Exp Clin Cancer Res. 2017 Jun 23;36:85. doi: 10.1186/s13046-017-0560-y (PMC5481925; doi:10.1186/s13046-017-0560-y)

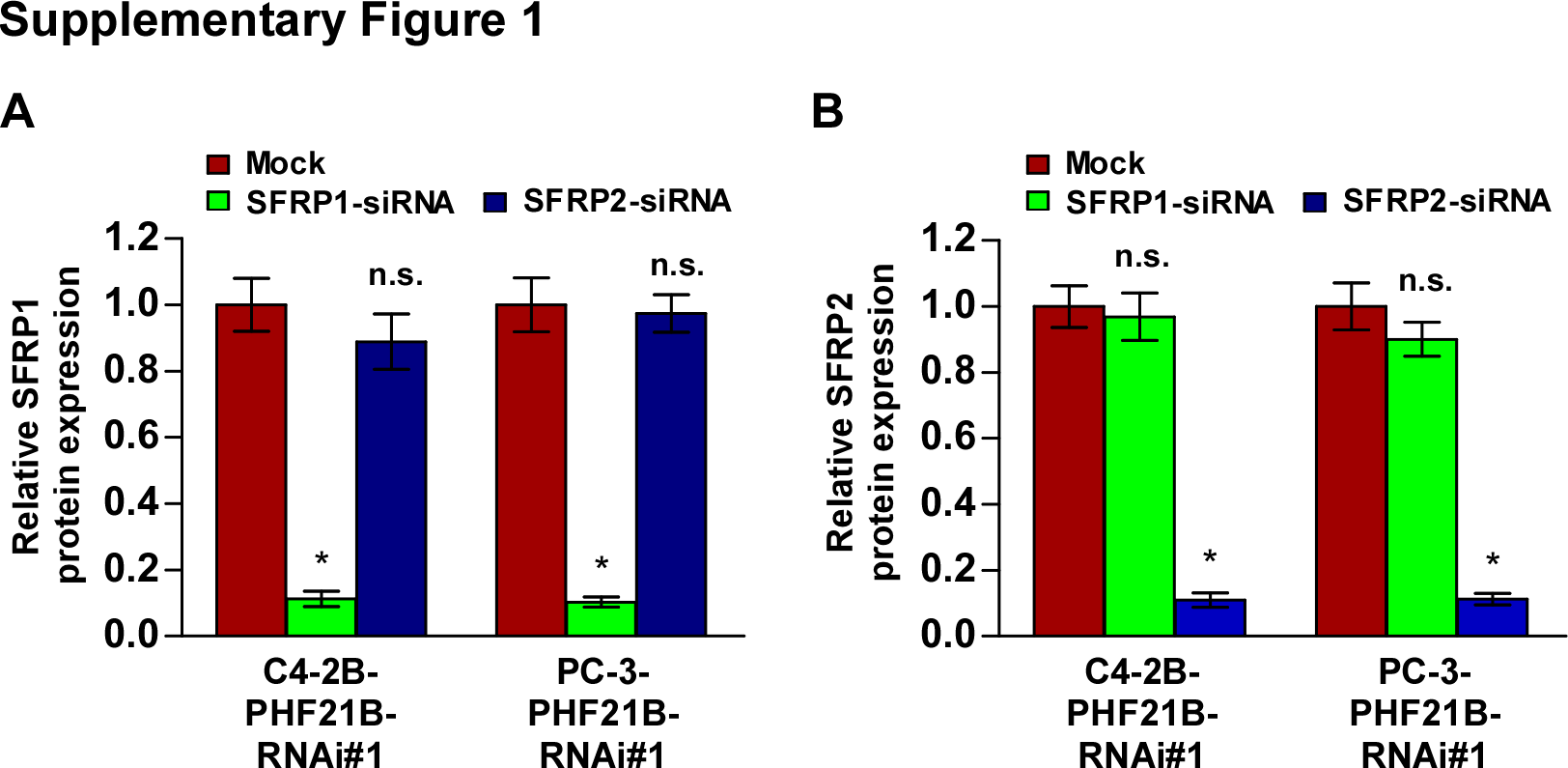

Supplement: Supplementary file 5 — SFRP1 and SFRP2 protein expression in the indicated cells. (A-B) Enzyme-linked immunosorbent assay (ELISA) analysis of protein levels of SFRP1 (A) and SFRP2 (B) in the supernatants of PHF21B-RNAi#1-C4-2B and -PC-3 cells treated with SFRP1 or SFRP2 siRNA. Error bars represent the means ± SD of 3 independent experiments. *P < 0.05. Not significant, n.s.. (TIF 103 kb) [file 13046_2017_560_MOESM5_ESM.tif]
